# Leveraging domain information to restructure biological prediction

**DOI:** 10.1186/1471-2105-12-S10-S22

**Published:** 2011-10-18

**Authors:** Xiaofei Nan, Gang Fu, Zhengdong Zhao, Sheng Liu, Ronak Y Patel, Haining Liu, Pankaj R Daga, Robert J Doerksen, Xin Dang, Yixin Chen, Dawn Wilkins

**Affiliations:** 1 Department of Computer and Information Science, University of Mississippi, USA; 2 Department of Medicinal Chemistry, University of Mississippi, USA; 3 Department of Mathematics, University of Mississippi, USA

## Abstract

**Background:**

It is commonly believed that including domain knowledge in a prediction model is desirable. However, representing and incorporating domain information in the learning process is, in general, a challenging problem. In this research, we consider domain information encoded by discrete or categorical attributes. A discrete or categorical attribute provides a natural partition of the problem domain, and hence divides the original problem into several non-overlapping sub-problems. In this sense, the domain information is useful if the partition simplifies the learning task. The goal of this research is to develop an algorithm to identify discrete or categorical attributes that maximally simplify the learning task.

**Results:**

We consider restructuring a supervised learning problem via a partition of the problem space using a discrete or categorical attribute. A naive approach exhaustively searches all the possible restructured problems. It is computationally prohibitive when the number of discrete or categorical attributes is large. We propose a metric to rank attributes according to their potential to reduce the uncertainty of a classification task. It is quantified as a conditional entropy achieved using a set of optimal classifiers, each of which is built for a sub-problem defined by the attribute under consideration. To avoid high computational cost, we approximate the solution by the expected minimum conditional entropy with respect to random projections. This approach is tested on three artificial data sets, three cheminformatics data sets, and two leukemia gene expression data sets. Empirical results demonstrate that our method is capable of selecting a proper discrete or categorical attribute to simplify the problem, i.e., the performance of the classifier built for the restructured problem always beats that of the original problem.

**Conclusions:**

The proposed conditional entropy based metric is effective in identifying good partitions of a classification problem, hence enhancing the prediction performance.

## Background

In statistical learning, a predictive model is learned from a hypothesis class using a finite number of training samples [1]. The distance between the learned model and the target function is often quantified as the generalization error, which can be divided into an approximation term and an estimation term. The former is determined by the capacity of the hypothesis class, while the latter is related to the finite sample size. Loosely speaking, given a finite training set, a complex hypothesis class reduces the approximation error but increases the estimation error. Therefore, for good generalization performance, it is important to find the right tradeoff between the two terms. Along this line, an intuitive solution is to build a simple predictive model with good training performance [2]. However, the “high dimensionality, small sample size” nature of many biological applications makes it extremely challenging to build a good predictive model: a simple model often fails to fit the training data, but a complex model is prone to overfitting. A commonly used strategy to tackle this dilemma is to simplify the problem itself using domain knowledge. In particular, domain information may be used to divide a learning task into several simpler problems, for which building predictive models with good generalization is feasible.

The use of domain information in biological problems has notable effects. There is an abundance of prior work in the field of bioinformatics, machine learning, and pattern recognition. It is beyond the scope of this article to supply a complete review of the respective areas. Nevertheless, a brief synopsis of some of the main findings most related to this article will serve to provide a rationale for incorporating domain information in supervised learning.

### Representation of domain information

Although there is raised awareness about the importance of utilizing domain information, representing it in a general format that can be used by most state-of-the-art algorithms is still an open problem [3]. Researchers usually focus on one or several types of application-specific domain information. The various ways of utilizing domain information are categorized as following: the choice of attributes or features, generating new examples, incorporating domain knowledge as hints, and incorporating domain knowledge in the learning algorithms [2].

Use of domain information in the choice of attributes could include adding new attributes that appear in conjunction (or disjunction) with given attributes, or selection of certain attributes satisfying particular criteria. For example, Lustgarten et al. [4] used the Empirical Proteomics Ontology Knowledge Bases in a pre-processing step to choose only 5% of candidate biomarkers of disease from high-dimensional proteomic mass spectra data. The idea of generating new examples with domain information was first proposed by Poggio and Vetter [5]. Later, Niyogi et al. [2] showed that the method in [5] is mathematically equivalent to a regularization process. Jing and Ng [6] presented two methods of identifying functional modules from protein-protein interaction (PPI) networks with the aid of Gene Ontology (GO) databases, one of which is to take new protein pairs with high functional relationship extracted from GO and add them into the PPI data. Incorporating domain information as hints has not been explored in biological applications. It was first introduced by Abu-Mostafa [7], where hints were denoted by a set of tests that the target function should satisfy. An adaptive algorithm was also proposed for the resulting constrained optimization.

Incorporating domain information in a learning algorithm has been investigated extensively in the literature. For example, the regularization theory transforms an ill-posed problem into a well-posed problem using prior knowledge of smoothness [8]. Verri and Poggio [9] discussed the regularization framework under the context of computer vision. Considering domain knowledge of transform invariance, Simard et al. [10] introduced the notion of transformation distance represented as a manifold to substitute for Euclidean distance. Schölkopf et al. [11] explored techniques for incorporating transformation invariance in Support Vector Machines (SVM) by constructing appropriate kernel functions. There are a large number of biological applications incorporating domain knowledge via learning algorithms. Ochs reviewed relevant research from the perspective of biological relations among different types of high-throughput data [12].

### Data integration

Domain information could be perceived of as data extracted from a different view. Therefore, incorporating domain information is related to integration of different data sources [13,14]. Altmann et al. [15,16] added prediction outcomes from phenotypic models as additional features. English and Butter [13] identified biomarker genes causally associated with obesity from 49 different experiments (microarray, genetics, proteomics and knock-down experiments) with multiple species (human, mouse, and worm), integrated these findings by computing the intersection set, and predicted previously unknown obesity-related genes by the comparison with the standard gene list. Several researchers applied ensemble-learning methods to incorporate learning results from domain information. For instance, Lee and Shatkay [17] ranked potential deleterious effects of single-nucleotide polymorphisms (SNP) by computing the weighted sum of various prediction results from four major bio-molecular functions, protein coding, splicing regularization, transcriptional regulation, and post-translational modification, with distinct learning tools.

### Incorporating domain information as constraints

Domain information could also be treated as constraints in many forms. For instance, Djebbari and Quackenbush [18] deduced prior network structure from the published literature and high-throughput PPI data, and used the deduced seed graph to generate a Bayesian gene-gene interaction network. Similarly, Ulitsky and Shamir [19] seeded a graphical model of gene-gene interaction from a PPI database to detect modules of co-expressed genes. In [6], Gene Ontology information was utilized to construct transitive closure sets from which the PPI network graph could grow. In all these methods, domain information was used to specify constraints on the initial states of a graph.

Domain information could be represented as part of an objective function that needs to be minimized. For example, Tian et al. [20] considered the measure of agreement between a proposed hypergraph structure and two domain assumptions, and encoded them by a network-Laplacian constraint and a neighborhood constraint in the penalized objective function. Daemen et al. [21] calculated a kernel from microarray data and another kernel from targeted proteomics domain information, both of which measure the similarity among samples from two angles, and used their sum as the final kernel function to predict the response to cetuximab in rectal cancer patients. Bogojeska et al. [22] predicted the HIV therapy outcomes by setting the model prior parameter from phenotypic domain information. Anjum et al. [23] extracted gene interaction relationships from scientific literature and public databases. Mani et al. [24] filtered a gene-gene network by the number of changes in mutual information between gene pairs for lymphoma subtypes.

Domain knowledge has been widely used in Bayesian probability models. Ramakrishnan et al. [25] computed the Bayesian posterior probability of a gene’s presence given not only the gene identification label but also its mRNA concentration. Ucar et al. [26] included ChIP-chip data with motif binding sites, nucleosome occupancy and mRNA expression data within a probabilistic framework for the identification of functional and non-functional DNA binding events with the assumption that different data sources were conditionally independent. In [27], Werhli and Husmeier measured the similarity between a given network and biological domain knowledge, and by this similarity ratio, the prior distribution of the given network structure is obtained in the form of a Gibbs distribution.

### Our contributions

In this article, we present a novel method that uses domain information encoded by a discrete or categorical attribute to restructure a supervised learning problem. To select the proper discrete/categorical attribute to maximally simplify a classification problem, we propose an attribute selection metric based on conditional entropy achieved by a set of optimal classifiers built for the restructured problem space. As finding the optimal solution is computationally expensive if the number of discrete/categorical attributes is large, an approximate solution is proposed using random projections.

## Methods

**Figure 1:**
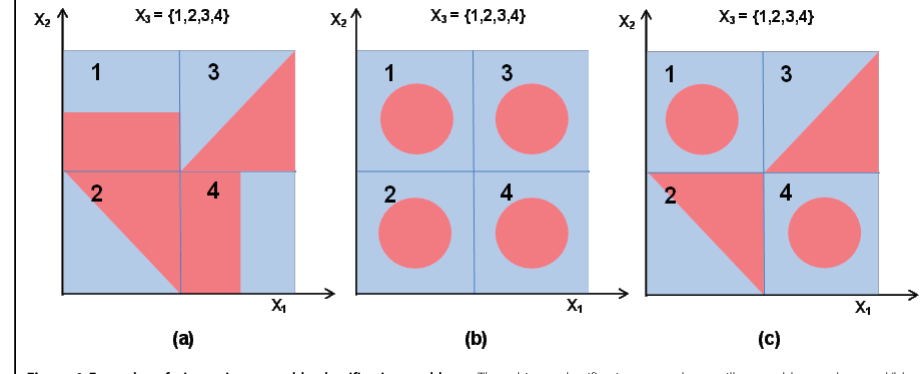
**Examples of piece-wise separable classification problems.** Three binary classification examples are illustrated here, where red/blue indicates positive/negative class. The figure shows that with the help of a categorical attribute *X*_3_, the three problems can be solved by simple hypothesis classes such as linear or polynomial models.

Many learning problems in biology are of high dimension and small sample size. The simplicity of a learning model is thus essential for the success of statistical modeling. However, the representational power of a simple model family may not be enough to capture the complexity of the target function. In many situations, a complex target function may be decomposed into several pieces, and each can be easily described using simple models. Three binary classification examples are illustrated in Figure 1, where red/blue indicates positive/negative class. In example (a), the decision boundary that separates two distinct color regions is a composite of multiple polygonal lines. It suggests the classification problem in (a) could not be solved by a simple hypothesis class such as a linear or polynomial model. Similarly, in examples (b) and (c), the decision boundary is so complex that neither a linear nor polynomial model can be fitted into these problems. Nevertheless, if the whole area is split into four different sub-regions (as shown in the figure, four quadrants marked from 1 to 4), the problem could be handled by solving each quadrant using a simple model individually. In example (a), the sub-problem defined on each quadrant is linearly separable. Likewise, each quadrant in (b) is suitable for a two-degree polynomial model. A linear model can be viewed as a special case of a two-degree polynomial. Therefore, the four sub-problems in (c) could be solved by a set of two-degree polynomial models. In the three examples, a categorical attribute *X*_3_ provides such partition information.

**Figure 2:**
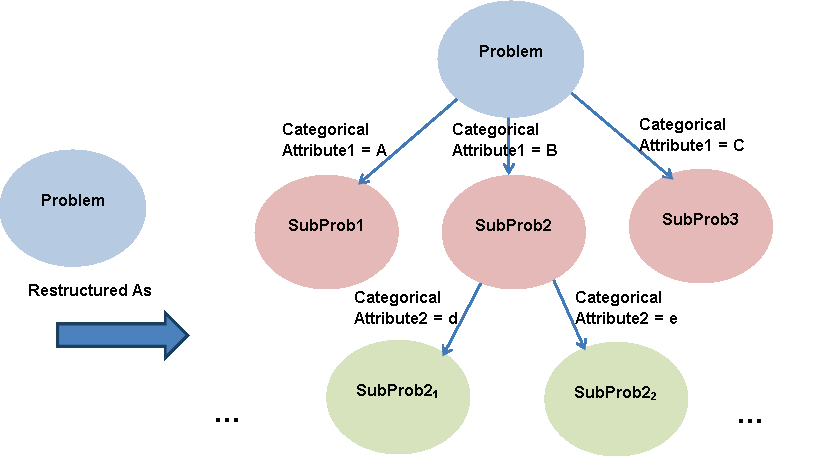
**Restructuring a problem by one or more categorical attribute.** By one or more discrete or categorical attributes, the original problem is split into multiple sub-problems. If the proper attribute is selected in the restructuring process, each sub-problem will have a comparably simpler target function.

Attributes like *X*_3_ exist in many biological applications. For instance, leukemia subtype domain knowledge, which can be encoded as a disicrete or categorical attribute, may help the prediction of prognosis. A discrete or categorical attribute provides a natural partition of the problem domain, and hence divides the original problem into several non-overlapping sub-problems. As depicted in Figure 2, the original problem is split into multiple sub-problems by one or more discrete or categorical attributes. If the proper attribute is selected in the restructuring process, each sub-problem will have a comparably simpler target function. Our approach is fundamentally different from the decision tree approach [28]: first, the tree-like restructuring process is to break up the problem into multiple more easily solvable sub-problems, not to make prediction decisions; second, the splitting criterion we propose here is based on the conditional entropy achieved by a categorical attribute and a hypothesis class, whereas the conditional entropy in decision trees is achieved by an attribute only. The conditional entropy will be discussed in detail later. Also, our method is related to feature selection in the sense that it picks categorical attributes according to a metric. However, it differs from feature selection in that feature selection focuses on the individual discriminant power of an attribute, and our method studies the ability of an attribute to increase the discriminant power of all the rest of the attributes. The categorical attributes selected by our method may or may not be selected by traditional feature selection approaches.

In theory, there’s no limit on the number of categorical attributes used in a partition if an infinite data sample is available. However, in reality, the finite sample size puts a limit on the number of sub-problems good for statistical modeling. In this article, we only consider incorporating one discrete or categorical attribute at a time. Identifying a discrete or categorical attribute that provides a good partition of a problem is nontrivial when the number of discrete or categorical attributes is large. In this paper, we propose a metric to rank these attributes.

### An attribute selection metric

A discrete or categorical attribute is viewed as having high potential if it provides a partition that greatly reduces the complexity of the learning task, or in other words, the uncertainty of the classification problem. A hypothesis class, such as the linear function family, is assumed beforehand. Therefore, we quantify the potential using the information gain achieved by a set of optimal classifiers, each of which is built for a sub-problem defined by the discrete or categorical attribute under consideration. Searching for the top ranked attribute with maximum information gain is equivalent to seeking the one with minimum conditional entropy. In a naive approach, an optimal prediction model is identified by comparing restructured problems using each discrete or categorical attribute. This exhaustive approach is computationally prohibitive when the number of discrete or categorical attributes is large. We propose to rank attributes using a metric that can be efficiently computed.

In a classification problem, consider a set of *l* samples (**x**, *y*) from an unknown distribution, **x** ∈ ℝ*^n^*, and *y* is the class label. In a *k*-class learning task, *y* gets a value from {1, …, *k*}; In a binary classification problem, *y* is either 1 or –1. *z* represents a discrete or categorical attribute with finite unique values. For simplicity, let’s assume *z* takes values from {1, …, *q*}, which offers a problem partition into *q* sub-problems, i.e. for all the samples when attribute *z* takes value *i*, *i* ∈ 1, …, *q. Z* is the set of all discrete and categorical attributes, *z* ∈ *Z.* A hypothesis class *M* is considered. We will first consider the linear model family. The metric can be generalized to a non-linear hypothesis class using the kernel trick [1].

For a binary classification problem, a linear discriminant function is formulated as *f*(**x**) = **w^T^x** + *c*, where **w** indicates the normal vector of the corresponding hyperplane and *c* is the offset parameter. For a multi-class task, if the one-vs-one method [29] is applied, there exists *k*(*k* – 1)/2 linear discriminant functions, each of which separates a pair of classes. Because a categorical attribute *z* divides the problem into *q* sub-problems, we define a model *m* for the whole problem as a set of linear discriminant functions on the *q* sub-problems: if it is a binary classification problem, *m* contains *q* linear discriminant functions; if it is a multi-class problem, *m* comprises *qk*(*k* – 1)/2 discriminant functions. Model *m* contains a pair of components (**w**, *c*), where **w** is the set of normal vectors of all of the discriminant functions in *m*, and *c* contains all of the linear function offset parameters in *m*.

The most informative attribute under the context discussed above is defined through the following optimization problem:

which is equivalent to

Note that the conditional entropy used here is fundamentally different from the one normally applied in decision trees. The traditional conditional entropy *H*(*y*|*z*) refers to the remaining uncertainty of class variable *y* given that the value of an attribute *z* is known. The conditional entropy used above is conditional on the information from attribute *z* and model *m*. In other words, the proposed method looks one more step ahead than a decision tree about data impurity of sub-problems.

### An approximated solution

The above optimization problem cannot be solved without knowledge of the probabilistic distribution of data. Sample version solutions may not be useful due to the curse of dimensionality: in high dimension feature spaces, a *finite number* of points may easily be separated by a hypothesis class (an infinitesimal conditional entropy), but the solution is more likely to be overfit than to be a close match to the target function. Taking a different perspective, if a categorical attribute is able to maximally simplify the learning task, the expected impurity value with respect to all possible models within the given hypothesis class should be small. This motivates the following approximation using the expected conditional entropy with respect to a random hyperplane:

The expectation could be estimated by the average over a finite number of trials. Hence, we randomly generate *N* sets of normal vectors (each set includes *q* normal vectors for binary-class or *qk*(*k* – 1)/2 for multi-class), search for the corresponding best offset for each normal vector, and calculate the average conditional entropy(1)

In the *i_th_* random projection, **w***_i_* includes all the normal vectors of the linear classifiers, each of which is built on a sub-problem, and *c_i_* does the same for the offsets. According to the definition of conditional entropy, *H*(*y*|*z*, (**w***_i_*, *c_i_*)) in (1) is formulated as:(2)

Probability *p*(*z* = *j*) is approximated by the sub-problem size ratio. The last step of the above derivation is based on the fact that the random projections are independent from the size of the sub-problems.

In a binary classification task, *z* = *j* denotes the *j_th_* sub-problem, and (**w***_ij_*, *c_ij_*) indicates the linear discriminant function of the *i_th_* random projection on the *j_th_* sub-problem. The discriminant function represented by (**w***_ij_*, *c_ij_*) classifies the *j_th_* sub-problem into two parts,  and :

*H*(*y*|*z* = *j*,**w***_ij_*, *c_ij_*) in (2) quantifies the remaining uncertainty of variable *y* in the *j_th_* sub-problem given the learned partition result  defined by the linear discriminant function with parameters (**w***_ij_*, *c_ij_*):(3)

In the computation of (3),  and .  and  are estimated by the proportion of positive/negative samples within  and , respectively.

In a multi-class setting, within a sub-problem, instead of two sub-regions (Ω^+^, Ω^–^), there are *q* sub-regions (Ω^1^, …, Ω*^q^*), each of which is the decision region for a class. All the categorical attributes are ranked according to (1).

### Extension to non-linear models

Our proposed metric could be easily extended to non-linear models using the kernel trick [1]. By the dual representation of a linear model, the normal vector is represented as a weighted summation of sample data.

where α*_I_* ∈ ℝ is a weight. The linear function is then formulated as:

Using the kernel trick, inner product  can be replaced by a kernel function *K. K*(**x***_i_*, **x**) is the inner product of **x***_i_* and **x** in the reproducing kernel Hilbert space. Therefore, the above linear discriminative function is transformed to,(4)

In our method, given a kernel *K*, random projections are achieved through *α_i_*.

## Results and discussion

We tested our method on three artificial data sets, three cheminformatics data sets and two cancer microarray data sets. The random projection was executed 1000 times for each data set.

Three different kernels were applied in this paper: linear, two-degree polynomial and Gaussian. The latter two kernels have one or more parameters. For the two-degree polynomial kernel, we used the default setting as *K*(**u**, **v**) = (**u^T^v**)^2^. Choosing a proper parameter *γ* in the Gaussian kernel *K*(**u**, **v**) = *exp*(– *γ*||**u** – **v**||^2^) is not an easy task. This paper focuses on how to select one (or more) categorical or discrete attribute(s) to divide the original problem into multiple simpler sub-problems. Selecting a proper model is not the theme of the work. Therefore, we list three Gaussian kernels using different *γ* values, 0.01, 1 and 10, to demonstrate that our restructuring process could be extended to non-linear models including the Gaussian kernel.

Many prediction problems have the property of small sample size and high dimensionality, for example, the learning tasks for the three cheminformatics data sets. Simple models under these circumstances are usually preferred. We applied a linear kernel on these three data sets, and analyzed the results from a cheminformaticist’s point of view. For the purpose of comparison, two-degree polynomial kernels and Gaussian kernels were also used.

The code was written with Matlab and libsvm package, and can be downloaded from http://cbbg.cs.olemiss.edu/StructureClassifier.zip.

### Artificial data sets

Three artificial data sets were generated to test our method using both linear and non-linear models. They are shown in Figure 1. Each artificial data is generated by four attributes: *X*_1_ and *X*_2_ are continuous attributes, and *X*_3_ and *X*_4_ are categorical attributes. The continuous attributes are uniformly distributed. *X*_3_ = {1, 2, 3, 4} denotes four different smaller square sub-regions. *X*_4_ = {1, 2} is a random categorical attribute for the purpose of comparison. In the experiment, we generated 10 sets for Artificial Data 1, 2, and 3, respectively. All 10 sets share the same values of attributes *X*_1_, *X*_2_, and *X*_3_, but *X*_4_ is random. Average results and standard deviations were computed.

The binary class information is coded by two distinct colors. Categorical attribute *X*_3_ provides interesting partitions: the partition in (a) leads to linear classification problems; the partition in (b) and (c) generates nonlinear problems that can be solved using techniques such as SVM with a polynomial kernel. Note that the original problem in (a) is not linear. The original problems in (b) and (c) are nonlinear, and not solvable using a polynomial kernel of degree 2.

Next, we assume linear classifiers in (a) and SVM with a polynomial kernel of degree 2 in (b) and (c). From Tables 1, 2, and 3, we see that the averaged estimated conditional entropy of *X*_3_ is always smaller than that of *X*_4_. Hence *X*_3_ is selected to restructure the problem. Next, we build both linear classifier and degree-2 polynomial SVM models on the original problem (we call it the baseline method), and linear and degree-2 polynomial models on the restructured problems introduced by *X*_3_. Significant improvements in both cross-validation (CV) accuracy and test accuracy are achieved using the partitions provided by *X*_3_. For comparison purposes, models were built on the restructured problem produced by *X*_4_. *X*_3_ outperforms *X*_4_ with a comfortable margin. There is no significant improvement using *X*_4_ than the baseline approaches.

**Table 1:** Experimental Results of Artificial Data 1 (Fig1 (a)) with Linear Model.

	Conditional Entropy	Training CV Accuracy(%)	Test Accuracy(%)
Baseline	–	59.6000 ± 3.2042	64.7750 ± 4.0285
*X*_3_	0.7860 ± 0.0044	99.5750 ± 0.2058	96.8607 ± 0.8680
*X*_4_	0.9001 ± 0.0035	61.1250 ± 1.7490	60.4881 ± 2.8090

**Table 2:** Experimental Results of Artificial Data 2 (Fig 1.(b)) Using Two-degree Polynomial Kernel.

	Conditional Entropy	Training CV Accuracy(%)	Test Accuracy(%)
Baseline	–	71.9750 ± 6.4737	71.0500 ± 7.9292
*X*_3_	0.8980 ± 0.0061	94.1000 ± 0.8350	94.3071 ± 0.9204
*X*_4_	0.9514 ± 0.0043	73.4000 ± 1.4443	73.8682 ± 2.8535

**Table 3:** Experimental Results of Artificial Data 3 (Fig 1.(c)) Using Two-degree Polynomial Kernel.

	Conditional Entropy	Training CV Accuracy(%)	Test Accuracy(%)
Baseline	–	73.1750 ± 5.7772	71.6025 ± 8.3302
*X*_3_	0.8455 ± 0.0059	96.5500 ± 0.8644	95.3658 ± 1.0224
*X*_4_	0.9328 ± 0.0032	72.8750 ± 1.5601	71.7689 ± 3.5528

### Cheminformatics data

We tested our approach on three cheminformatics data sets, biological activity data of glycogen synthase kinase-3*β* inhibitors, cannabinoid receptor subtypes CB1 and CB2 activity data, and CB1/CB2 selectivity data.

#### Biological activity prediction of glycogen synthase kinase-3*β* inhibitors

In the first dataset, data samples (IC50) were collected from several publications, with a range from subnanomolar to hundred micromolar. The biological activities have been discretized as binary values: highly active and weakly active, with a cut-off value of 100 nM. The aim is to predict biological activity based on physicochemical properties and other molecular descriptors of the compounds calculated using DragonX software [30]. This data set was divided into 548 training samples and 183 test samples. The attribute set size is 3225, among which 682 are categorical attributes. Some discrete attributes contain a large number of values. For a fixed sized training set, some regions generated by a partition using such attributes may contain a very small number of samples (many times 1 or 2), and hence are not suitable for training a classifier. So we filtered out attributes with more than 10 unique values.

**Figure 3:**
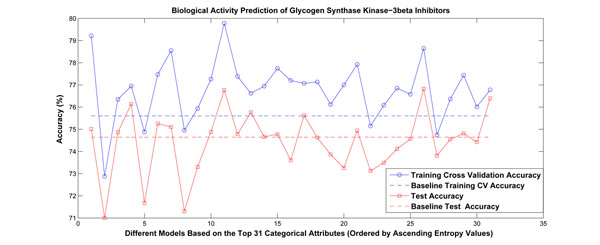
**Experimental results for biological activity prediction of glycogen synthase kinase-3*β* inhibitors.** The categorical attributes were ranked based on their estimated conditional entropies. We chose the first 31 attributes with smallest entropy values for problem partition. We restructured the learning problem according to these candidate attributes separately, and built linear models for each partition. Among the 31 attributes, there are 17 categorical attributes whose performance beat the baseline approach in terms of both cross-validation accuracy and test accuracy.

Using a linear kernel, we ranked the categorical attributes based on their estimated conditional entropies. The top 31 attributes (with smallest estimated conditional entropy) were viewed as candidate attributes for problem partition. We restructured the learning problem according to these candidate attributes separately, and built linear models for each partition. Figure 3 shows the experimental results. Among the 31 attributes, there are 17 categorical attributes whose performance beat the baseline approach in terms of both cross-validation accuracy and test accuracy. The detailed performance values and the names of the attributes are provided in Table 4. Compared with linear kernels, the ranking orders of these attributes by two-degree polynomial and Gaussian kernels and their corresponding cross-validation and test accuracies are provided in Table 5 as well. For Gaussian kernels, we notice performance improvement for most of the selected attributes under all three tested *γ* values. The highest performance was achieved when the Bioassay Protocol attribute was selected to restructure the problem. This attribute records the different protocols used during the cheminformatics experiment, and also indicates distinct chemotypes.

**Table 4:** Learning Performance for the Selected Categorical Attributes in Biological Activity Data of Glycogen Synthase Kinase-3*β* Inhibitors Using Linear Kernel.

	Entropy list order	Training CV Accuracy(%)	Test Accuracy(%)
Baseline	–	75.60	74.64
nCIR	1	79.21	75.01
F06[N-O]	2	76.35	74.86
H-049	3	76.95	76.14
nN	7	77.38	74.78
F04[N-N]	8	78.55	75.10
Bioassay Protocol	9	79.78	76.76
nHDon	12	77.26	74.88
H-050	13	77.26	74.88
nDB	15	77.74	74.78
F07[C-Br]	16	76.62	75.76
F02[N-O]	22	77.07	75.62
N-075	23	78.65	76.83
F06[C-Br]	25	76.94	74.66
F02[N-N]	26	77.93	74.92
N-074	30	76.78	76.39
F03[N-N]	31	77.44	74.81

**Table 5:** Performance Comparison for the Selected Categorical Attributes in Biological Activity Data of Glycogen Synthase Kinase-3*β* Inhibitors Using Two-degree Polynomial Kernel and Gaussian Kernels.

	Entropy list order				Training CV Accuracy(%)				Test Accuracy(%)			
	Poly	Gausssian			Poly	Gausssian (*γ*)			Poly	Gausssian (*γ*)		
		0.01	1	10		0.01	1	10		0.01	1	10

Baseline	–	–	–	–	76.23	73.10	62.74	59.42	74.26	70.69	60.58	57.44
nCIR	3	2	1	1	78.84	75.41	64.48	60.15	74.55	71.23	61.26	58.02
F06[N-O]	2	1	2	2	77.62	73.23	63.34	60.23	73.28	70.49	60.87	56.95
H-049	4	5	4	4	79.75	74.69	65.18	61.03	75.14	71.87	62.76	57.26
nN	1	6	6	7	79.24	74.87	64.77	60.49	75.23	71.04	62.38	57.15
F04[N-N]	7	3	5	6	78.32	74.14	63.14	60.63	74.16	70.02	61.79	57.69
Bioassay Protocol	8	7	3	5	79.15	75.54	65.15	62.25	76.03	72.87	63.76	59.34
nHDon	11	19	18	19	77.63	74.18	63.05	60.02	75.12	71.17	60.34	57.28
H-050	21	7	7	9	76.95	73.57	63.72	60.35	74.34	71.09	59.28	56.94
nDB	13	24	21	25	75.37	73.89	62.83	59.25	73.22	70.18	60.47	56.74
F07[C-Br]	17	12	15	16	77.25	74.58	63.04	60.42	73.96	71.65	61.07	58.15
F02[N-O]	25	16	13	15	76.14	73.87	62.95	58.72	72.87	70.66	60.84	57.35
N-075	20	17	17	21	78.06	74.92	63.74	60.87	75.64	71.29	62.88	59.04
F06[C-Br]	27	26	25	23	75.44	72.05	61.43	58.28	72.76	69.96	60.03	55.74
F02[N-N]	33	30	26	32	77.83	74.15	63.82	60.96	74.56	70.75	61.44	59.45
N-074	29	35	33	34	76.54	73.47	63.95	60.42	74.75	71.03	60.58	57.96
F03[N-N]	36	31	34	37	75.69	74.26	62.65	59.35	73.48	70.33	59.87	57.28

The highest cross-validation performance attribute, nCIR, belongs to the constitutional descriptors. Constitutional descriptors reflect the chemical composition of a compound without the structural information of the connectivity and the geometry. nCIR means the number of circuits, which includes both rings and the larger loop around two or more rings. For instance, naphthalene contains 2 rings and 3 circuits. This attribute could easily distinguish ring-containing structures and linear structures. Many attributes selected have names starting with “F0”. They are from the 2D frequency fingerprints, which define the frequency of specific atom pairs at different topological distances from 1 to 10. Among all of the 2D frequency fingerprints, the atom pair “N-N” appeared multiple times. The frequency of this atom pair at different topological distances from 2 to 4 could be used to separate the dataset. Another important atom pair is “N-O”, which also appeared multiple times in the list. Both atom pairs contain the nitrogen atom which is highly common in the kinase inhibitor structures, since it plays a key role in the hydrogen bond interactions between the inhibitor and the kinase. Another atom-centered fragment attribute is H-049, which means the atom H attached to any of C^3^(sp^3^) / C^2^(sp^2^) / C^3^(sp^2^) / C^3^(sp) groups. The superscripts on the carbons stand for the formal oxidation number and the contents in the parentheses stand for the hybridization state. The hydrogen in an H-049 fragment has negative atomic hydrophobicity and low molecular refractivity [31], so they are less hydrophobic and more hydrophilic. H-049 could be used to separate the database because the kinase inhibitors are usually hydrophilic in order to bind to the protein in the ATP-binding pocket.

#### Cannabinoid receptor subtypes CB1 and CB2 activity and selectivity prediction

**Figure 4:**
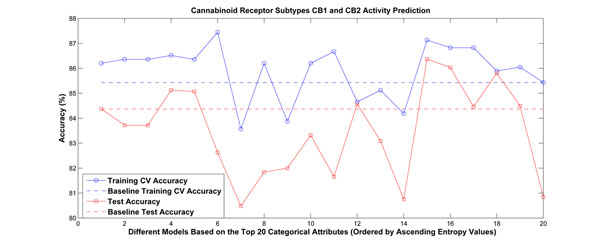
**Experimental results for cannabinoid receptor subtypes CB1 and CB2 activity prediction.** The categorical attributes were ranked based on their estimated conditional entropies, and the top 20 attributes were chosen to partition the problem separately. Linear models were built for each partition. Among the 20 attributes, there are 8 having better performance than the baseline approach in terms of both cross-validation accuracy and test accuracy.

**Figure 5:**
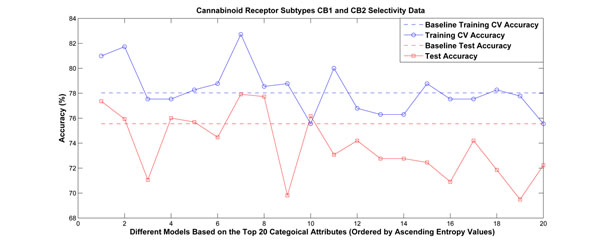
**Experimental results for cannabinoid receptor subtypes CB1 and CB2 selectivity prediction.** The categorical attributes were ranked based on their estimated conditional entropies, and the top 20 attributes were choseN to partition the problem separately. Linear models were built for each partition. Among the 20 attributes, there are 5 having better performance than the baseline approach in terms of both cross-validation accuracy and test accuracy.

The second and third data sets are for cannabinoid receptor subtypes CB1 and CB2. They were also computed from DragonX software, and have 3225 attributes. The second data set is to predict activity and was divided into 645 training samples and 275 test samples. It contains 683 categorical attributes. The third set is to predict selectivity of binding to CB1 vs. CB2 and includes 405 training samples, 135 test samples, and 628 categorical attributes. The experimental results are shown in Figures 4 and 5, respectively. We ordered the categorical attributes based on their conditional entropy values in ascending order. Note that the model based on the first attribute always performed better than the baseline approach.

The classes and descriptions for the attributes that result in better performance than the baseline approach are listed in Tables 6 and 7. The learning performance comparison with other non-linear kernels are shown in Tables 8 and 9 respectively. For the CB activity, among the eight features, six of them (F01[N-O], N-076, nArNO2, B01[N-O], N-073 and nN(CO)2) involve nitrogen. This clearly suggests that nitrogen plays a significant role in classifying the active CB ligands. The input data showed that the values of N-076 and nArNO2 for all the active compounds are 0. Hence, it is very likely that any compound with the Ar-NO2 / R–N(–R)–O / RO-NO moiety or a nitro group may not be active. In addition, the majority of the active compounds have F01[N-O] and nN(CO)2 values of 0. Hence, the lack of a N-O or an imide moiety is perhaps a common feature of active CB ligands. Furthermore, the N-073 feature is distributed between 0 and 2 in the active compounds. Hence, the nitrogen atom in the active compounds, if it exists, may appear in the form of Ar2NH / Ar3N / Ar2N-Al / R..N..R. Its role may include acting as a hydrogen bond acceptor, or affecting the polarity of the molecule, which may facilitate the ligand binding. For the CB selectivity problem, two features (nDB and nCconj) involve double bonds. Both of these address the non-aromatic C=C double bond and the values are primarily distributed between 0 - 6 and 0 - 2, respectively, in the selective compounds. The role of this bond, if it exists, is perhaps to form hydrophobic interactions with the proteins. It is also interesting to note that the nCconj attribute leads to the best test accuracy for both the activity and selectivity datasets. The descriptions of selected categorical attributes can be viewed in Tables 10 and 11.

**Table 6:** Learning Performance for the Selected Categorical Attributes in Cannabinoid Receptor Subtypes CB1 and CB2 Activity Data Using Linear Model.

	Entropy list order	Training CV Accuracy(%)	Test Accuracy(%)
Baseline	–	85.43	84.36
F01[N-O]	1	86.20	84.37
N-076	4	86.51	85.12
nArNO2	5	86.36	85.07
nCconj	15	87.13	86.37
C-034	16	86.82	86.04
B01[N-O]	17	86.82	84.46
N-073	18	85.89	85.81
nN(CO)2	19	86.05	84.49

**Table 7:** Learning Performance for the Selected Categorical Attributes in Cannabinoid Receptor Subtypes CB1 and CB2 Selectivity Data Using Linear Model.

	Entropy list order	Training CV Accuracy(%)	Test Accuracy(%)
Baseline	–	78.02	75.56
O-058	1	80.99	77.35
nDB	2	81.73	75.94
F06[C-Cl]	5	78.27	75.63
nCconj	7	82.72	77.92
C-026	8	78.55	77.73

**Table 8:** Performance Comparison for the Selected Categorical Attributes in Cannabinoid Receptor Subtypes CB1 and CB2 Activity Data Using Two-degree Polynomial Model and Gaussian Models.

	Entropy list order				Training CV Accuracy(%)				Test Accuracy(%)			
	Poly	Gausssian			Poly	Gausssian			Poly	Gausssian		
		*γ* = 0.01	*γ* =1	*γ* = 10		*γ* = 0.01	*γ* = 1	*γ* = 10		*γ* = 0.01	*γ* = 1	*γ* = 10

Baseline	–	–	–	–	86.51	75.34	65.21	66.76	85.58	74.35	65.79	65.61
F01[N-O]	1	2	1	1	85.15	76.12	66.16	66.44	85.44	76.14	65.65	66.15
N-076	4	5	4	4	87.50	77.05	66.98	67.33	86.12	76.89	66.34	66.79
nArNO2	6	7	5	5	86.82	75.14	66.78	66.58	85.27	84.35	76.34	64.96
nCconj	16	14	10	12	86.61	77.12	67.03	66.79	83.31	76.72	63.77	65.74
C-034	17	16	11	17	85.98	76.38	66.44	65.89	85.69	75.28	64.59	65.88
B01[N-O]	20	19	19	18	87.21	76.38	66.38	66.66	86.72	76.37	66.29	65.62
N-073	21	20	21	21	84.96	74.79	65.02	65.26	84.15	75.34	64.45	63.71
nN(CO)2	23	24	27	25	86.77	73.72	66.05	64.37	85.78	73.22	63.76	62.96

**Table 9:** Performance Comparison for the Selected Categorical Attributes in Cannabinoid Receptor Subtypes CB1 and CB2 Selectivity Data Using Two-degree Polynomial Model and Gaussian Models.

	Entropy list order				Training CV Accuracy(%)				Test Accuracy(%)			
	Poly	Gausssian			Poly	Gausssian			Poly	Gausssian		
		*γ* = 0.01	*γ* = 1	*γ* = 10		*γ* = 0.01	*γ* = 1	*γ* = 10		*γ* = 0.01	*γ* = 1	*γ* = 10

Baseline	–	–	–	–	76.04	67.15	57.28	57.67	74.89	65.12	54.84	53.33
O-058	2	1	2	2	79.92	70.12	60.34	79.12	65.96	56.34	56.02	53.21
nDB	3	3	4	4	80.05	71.34	61.22	80.36	76.32	67.78	57.67	55.32
F06[C-Cl]	7	8	7	8	79.73	69.96	58.27	79.12	75.12	63.29	54.79	53.29
Cconj	6	7	8	7	78.75	67.54	57.65	77.64	76.07	65.96	55.36	54.34
C-026	9	10	9	11	77.96	68.32	57.34	58.12	75.48	65.32	54.96	53.69

**Table 10:** Descriptions for the Selected Categorical Attributes in Cannabinoid Receptor Subtypes CB1 and CB2 Activity Data.

	Attribute Class	Description
F01[N-O]	2D frequency fingerprints	frequency of N-O at topological distance 1
N-076	Atom-centered fragments	Ar-NO2 / R–N(–R)–O / RO-NO
nArNO2	Functional group counts	number of nitro groups (aromatic)
nCconj	Functional group counts	number of non-aromatic conjugated C(*sp2*)
C-034	Atom-centered fragments	R–CR..X
B01[N-O]	2D binary fingerprints	presence/absence of N-O at topological distance 1
N-073	Atom-centered fragments	Ar_2_NH / Ar_3_N / Ar_2_N-Al / R..N..R
nN(CO)2	Functional group counts	number of imides (thio-)-C(=Y1)-N(Y)-C(=Y1)- Y=H or C, Y1= O or S

R represents any group linked through carbon; X represents any electronegative atom (O, N, S, P, Se, halogens); Al and Ar represent aliphatic and aromatic groups, respectively; = represents a double bond; – represents an aromatic bond as in benzene or delocalized bonds such as the N-O bond in a nitro group; .. represents aromatic single bonds as in the C-N bond in pyrrole.

**Table 11:** Descriptions for the Selected Categorical Attributes in Cannabinoid Receptor Subtypes CB1 and CB2 Selectivity Data.

	Attribute Class	Description
O-058	Atom-centered fragments	=O
nDB	Constitutional descriptors	number of double bonds
F06[C-Cl]	2D frequency fingerprints	frequency of C-Cl at topological distance 6
nCconj	Functional group counts	number of non-aromatic conjugated C(*sp2*)
C-026	Atom-centered fragments	R–CX..R

R represents any group linked through carbon; X represents any electronegative atom (O, N, S, P, Se, halogens); Al and Ar represent aliphatic and aromatic groups, respectively; = represents a double bond; – represents an aromatic bond as in benzene or delocalized bonds such as the N-O bond in a nitro group; .. represents aromatic single bonds as in the C-N bond in pyrrole.

### Leukemia gene data

The two leukemia gene data sets used are defined in Yeoh et al. [32] and Golub et al. [33], respectively. We applied a linear classifier, SVM with a two-degree polynomial kernel and Gaussian kernels on these two data sets.

Yeoh’s data [34] comprises gene expression data and two additional categorical attributes, Subtype and Protocol. Subtype indicates specific genetic subtypes of Acute lymphoblastic leukemia (ALL), and Protocol means distinct therapies. The entire set contains 201 continuous complete remission (CCR) samples and 32 relapse cases (including 27 Heme relapses and 5 additional relapses). We randomly split the data into training and test sets with 174 and 59 samples, respectively. The original data contains 12627 attributes, which is almost two orders of magnitude larger than the training set size. We used the 58 preselected attributes provided in the original paper and two additional categorical attributes to predict prognosis. Tables 12, 13, and 14 show the experimental results using linear, two-degree polynomial and Gaussian kernels, respectively. The subtype categorical attribute has smaller estimated conditional entropy than Protocol, and is thus selected to divide the problem. The learning performances from both the linear model and SVM demonstrate that it is the right choice.

**Table 12:** Experimental Results of ALL Prognosis Prediction Using Preselected Attribute Sets and Linear Model.

	Conditional Entropy	Training CV Accuracy(%)	Test Accuracy(%)
Baseline	–	85.06	89.83
Subtype	0.3659	89.08	92.20
Protocol	0.5616	85.06	89.96

**Table 13:** Experimental Results of ALL Prognosis Prediction Using Preselected Attribute Sets and Two-degree Polynomial Kernel.

	Conditional Entropy	Training CV Accuracy(%)	Test Accuracy(%)
Baseline	–	85.06	89.83
Subtype	0.3638	89.08	92.20
Protocol	0.5630	86.78	87.46

**Table 14:** Experimental Results of ALL Prognosis Prediction Using Preselected Attribute Sets and Gaussian Kernel.

	Conditional Entropy			Training CV Accuracy(%)			Test Accuracy(%)		
	*γ* = 0.01	*γ* =1	*γ* = 10	*γ* = 0.01	*γ* =1	*γ* = 10	*γ* = 0.01	*γ* =1	*γ* = 10

Baseline	–	–	–	85.06	85.06	85.06	89.83	89.83	89.83
Subtype	0.5656	0.5662	0.5662	88.51	88.51	88.51	92.20	92.20	92.20
Protocol	0.3829	0.3835	0.3840	85.06	85.06	85.06	89.96	89.96	89.96

Golub’s data set [35] includes gene expression data and four categorical attributes, BM/PM, T/B-cell, FAB, and Gender. A random split was used to separate the whole data set into 54 training samples and 18 test samples. Correlation-based Feature Selection [36] was executed beforehand to decrease the attribute dimension from 7133 to 45. The 45 attributes include two categorical attributes, T/B-cell and FAB. FAB denotes one of the most commonly used classification schemata for Acute Myeloid Leukemia (AML). BM/PM and Gender had been deleted during the feature selection process. The goal is to predict ALL or AML. From Tables 15, 16 and 17, we can see that both T/B-cell and FAB have very small conditional entropy values (it may be because it is an easy learning problem). The T/B-cell categorical attribute was selected to partition the problem.

**Table 15:** Experimental Results of ALL/AML Prediction Using Attributes Selected by CFS and Linear Model.

	Conditional Entropy	Training CV Accuracy(%)	Test Accuracy(%)
Baseline	–	100.00	99.50
T/B-cell	7.1491e-16	100.00	100.00
FAB	1.1666e-15	100.00	99.70

**Table 16:** Experimental Results of ALL/AML Prediction Using Attributes Selected by CFS and Two-degree Polynomial Kernel.

	Conditional Entropy	Training CV Accuracy(%)	Test Accuracy(%)
Baseline	–	100.00	94.44
T/B-cell	7.1491e-16	100.00	100.00
FAB	1.1666e-15	100.00	100.00

**Table 17:** Experimental Results of ALL/AML Prediction Using Attributes Selected by CFS and Gaussian Kernel.

	Conditional Entropy			Training CV Accuracy(%)			Test Accuracy(%)		
	*γ* = 0.01	*γ* =1	*γ* = 10	*γ* = 0.01	*γ* =1	*γ* = 10	*γ* = 0.01	*γ* =1	*γ* = 10

Baseline	–	–	–	68.52	64.81	64.81	66.67	66.67	66.67
Subtype	7.1491e-16	7.1491e-16	7.1491e-16	100.00	100.00	100.00	100.00	100.00	100.00
Protocol	1.1666e-15	1.1666e-15	1.1666e-15	100.00	100.00	100.00	100.00	100.00	100.00

### Discussions and future work

For choosing a proper partition attribute, we could either select the one with the smallest conditional entropy, or the one with the highest training cross-validation accuracy among multiple candidates. The first strategy worked well for all the data sets — while it may not provide the best performing partition, it always outperformed the baseline. The second strategy yielded the best answer for most cases — glycogen synthase kinase-3*β* inhibitors data is an example — however, it failed on cannabinoid receptor subtypes CB1 and CB2 activity data.

In addition to simplifying the learning problem, the selected categorical attribute may provide additional perspective in unveiling hidden biological information. For example, the attributes chosen from cannabinoid receptor subtypes CB1 and CB2 data sets supply useful information for compound design.

Although the restructuring process organizes classifiers in a tree, it is fundamentally different from the splitting process of a standard decision tree: the conditional entropy in the proposed metric depends on a classifier family. In the future, we would like to extend the restructuring process to multiple layers using one or more attributes.

## Conclusions

We propose a method of restructuring a supervised learning problem using a discrete/categorical attribute. Such attributes naturally divide the original problem into several non-overlapping sub-problems. With a proper choice of the attribute, the complexity of the learning task is reduced, and the prediction performance enhanced. Selecting a proper discrete or categorical attribute that maximally simplifies the learning task is a challenging problem. A naive approach requires exhaustive searching for the optimal learning model for each possible restructured problem, and hence is computationally prohibitive. We propose a metric to select the categorical attribute based on the estimated expected conditional entropy with respect to random projections. This method can be applied to multi-class and non-linear problems. Experimental results demonstrate the good performance of the proposed approach on several data sets. Future work is to develop methods/metrics to extend the approach to efficiently identify multiple categorical attributes for problem restructuring.

## Competing interests

All the authors declare that they have no competing interests.

## Authors' contributions

XN, GF, ZZ, SL, RJD, YC, and DW contributed to the method proposal. Dang helped in the method formulation part. GF and RJD were responsible for preparing the biological activity data of glycogen synthase kinase-3 inhibitors and result interpretation. RYP, HL, PRD, and RJD contributed to the preparation of cannabinoid receptor subtype CB1 and CB2 activity and selectivity data sets and result interpretation. XN was responsible for the programming and collecting of other data sets, and completed the writing with assistance from the other authors.
